# Pituitary apoplexy presenting as refractory hyponatremia

**DOI:** 10.1210/jcemcr/luag052

**Published:** 2026-04-22

**Authors:** Samira Chandra, Thomas Banh, Soumya Thumma, Shrikant Tamhane, Luis Augusto Juncos

**Affiliations:** Department of Medicine, Baptist Health - UAMS Internal Medicine Residency Program, North Little Rock, AR 72117, USA; Department of Medicine, Baptist Health - UAMS Internal Medicine Residency Program, North Little Rock, AR 72117, USA; Division of Endocrinology, Baptist Health - UAMS Internal Medicine Residency Program, North Little Rock, AR 72117, USA; Division of Endocrinology, Baptist Health - UAMS Internal Medicine Residency Program, North Little Rock, AR 72117, USA; FME Global Medical Office, Medical Affairs, Fresenius Medical Care, Waltham, MA 02451, USA

**Keywords:** pituitary apoplexy, hyponatremia, SIADH, syndrome of inappropriate antidiuretic hormone secretion, adrenal insufficiency, hypocortisolism, electrolyte free water clearance

## Abstract

A previously healthy 24-year-old man presented with subacute worsening headache. Initial evaluation revealed euvolemic hypotonic hyponatremia and central hypothyroidism. Brain magnetic resonance imaging (MRI) demonstrated a sellar lesion concerning for a Rathke cleft cyst vs hemorrhagic macroadenoma. His hyponatremia was initially attributed to syndrome of inappropriate antidiuretic hormone and treated with fluid restriction and hypertonic saline. However, his hyponatremia and neurologic symptoms worsened. Given the patient's headache and sellar pathology, pituitary apoplexy was considered. A pituitary panel was obtained and empiric hormone replacement therapy initiated with IV hydrocortisone (100 mg every 6 hours) and levothyroxine (100 mcg daily). His symptoms and laboratory abnormalities improved to near-complete resolution within 72 hours. Pituitary testing confirmed panhypopituitarism. The patient was discharged on levothyroxine and a hydrocortisone taper. A follow-up pituitary MRI demonstrated interval reduction in lesion size. At 6-month follow-up, recovery of the pituitary axes was observed except for residual GH deficiency. Pituitary apoplexy is a medical emergency requiring prompt recognition and initiation of hormone replacement therapy. Secondary adrenal failure may cause hyponatremia that is refractory to standard therapy, thus mandating its consideration in this setting.

## Introduction

Hyponatremia is a common electrolyte abnormality resulting from a relative excess of water in relation to sodium [1]. It is an independent predictor of morbidity and mortality with clinical manifestations that vary according to the severity and rate of sodium decline. Mild to moderate hyponatremia causes nonspecific neurologic symptoms including headache, nausea, vomiting, weakness, confusion, and gait instability, whereas severe or rapidly progressing hyponatremia may result in seizures, coma, and other life-threatening complications, thus constituting a medical emergency.

Among the causes of euvolemic hyponatremia, syndrome of inappropriate antidiuretic hormone (SIADH) is the most common in hospitalized patients, whereas pituitary apoplexy is rare [1-3]. Pituitary apoplexy is an endocrine emergency resulting from hemorrhage or infarction of the pituitary gland, with or without a preexisting adenoma. Typical features include headache, visual field defects, multiple pituitary hormone deficiencies, and associated metabolic abnormalities. Because manifestations of hypopituitarism overlap with those of hyponatremia, pituitary dysfunction may be misattributed to other etiologies (eg, SIADH), leading to refractory hyponatremia and clinical deterioration. We report a case of pituitary apoplexy presenting with refractory hyponatremia, underscoring the need for timely and through evaluation of patients with atypical or refractory hyponatremia.

## Case presentation

A 24-year-old male without significant past medical history presented with a headache of 12 days duration. He described it as a continuous, dull, moderately intense ache, worsened by leaning forward and associated with nausea. He had presented twice to the emergency department and once to urgent care with these symptoms. Because he was normotensive, had unremarkable laboratory values, and had a noncontrast head computed tomography scan that was negative, he was treated symptomatically with analgesics and discharged after each visit. Persistent symptoms prompted reevaluation and hospital admission.

## Diagnostic assessment

The patient’s blood pressure was 151/91 mmHg and heart rate 74 beats/min. Physical examination was unremarkable, and he appeared euvolemic. Laboratory testing showed hyponatremia with plasma sodium 123 mEq/L [International System of Units: 123 mmol/L) (reference range: 136-145 mEq/L; International System of Units: 136-145 mmol/L), low plasma osmolality 260 mOsm/kg (260 mmol/kg) (reference range 270-295 mOsm/kg; 270-295 mmol/kg), inappropriately elevated urine osmolarity 570 mOsm/kg (570 mmol/kg) (80-1200 mOsm/kg; 80-1200 mmol/kg), and normal plasma uric acid 5.3 mg/dL (0.31 mmol/L) (reference range 3.5-7.2 mg/dL; 0.21–0.43 mmol/L). Thyroid studies demonstrated low TSH 0.2 mIU/mL (0.2 mIU/L) (0.35-4.94 mIU/L; 0.35-4.94 mIU/L), and low-normal free T4 0.82 ng/dL (10.6 pmol/L) (0.70-1.48 ng/dL; 9.03-18.1 pmol/L). Based on these findings, the hyponatremia was attributed primarily to SIADH. A repeat noncontrast computed tomography scan of the head was again unremarkable.

## Treatment

The patient was placed on fluid restriction (1 L/day). The following day, he reported worsening headache, new-onset dizziness, muscle cramps, rigors, and increased urinary frequency. Plasma sodium had declined to 120 mEq/L (120 mmol/L). Urinalysis was unremarkable, with specific gravity 1.017 and pH 6.0. Urine electrolytes demonstrated inappropriately elevated sodium 55 mEq/L (55 mmol/L), potassium 29 mEq/L (29 mmol/L), and chloride 63 mEq/L (63 mmol/L) (no reference range for all electrolytes).

**Figure 1:**
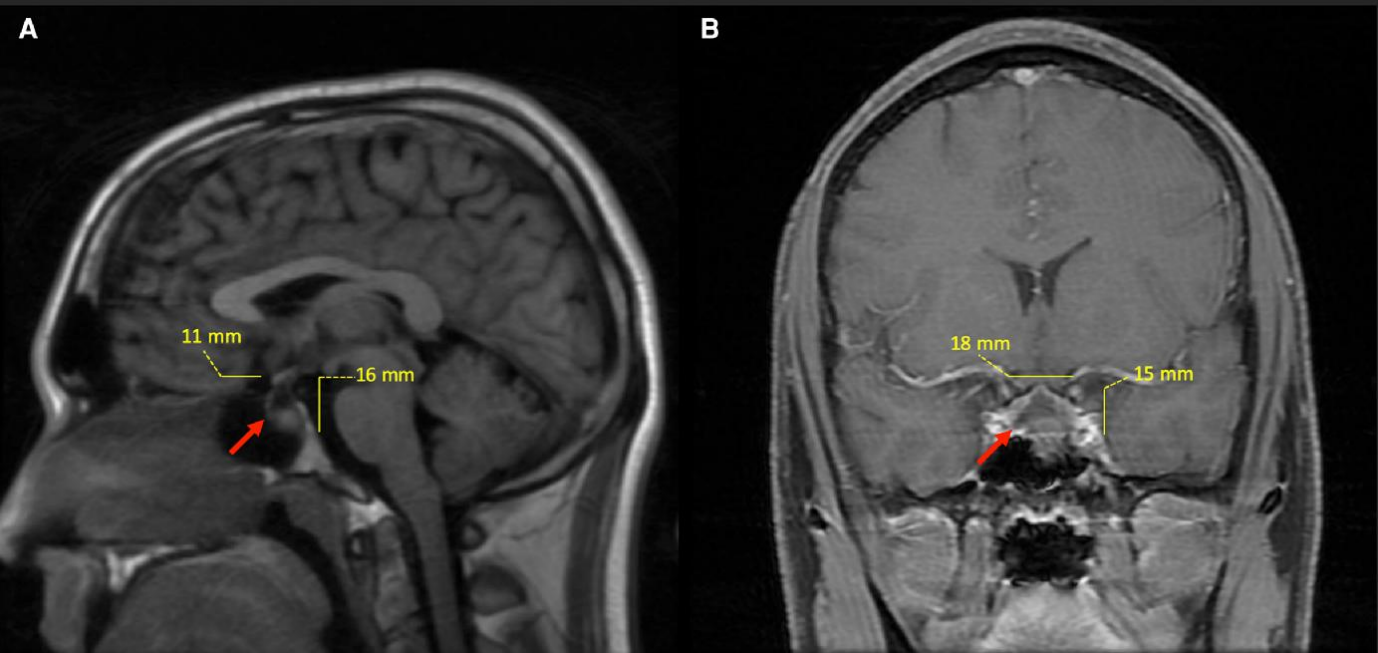
Initial magnetic resonance imaging of the brain with and without IV contrast. (A) Sagittal T1 flair view. (B) Coronal with contrast and fat-saturated view. There is heterogenous composition of the Rathke cleft cyst (identified by arrows in Fig. 1A). The cyst does have a significant size of 1.1 × 1.8 × 1.5 cm in the anteroposterior, transverse, and craniocaudal dimensions, but it does not cause mass effect (Fig. 1B).

Given worsening hyponatremia, he was transferred to the intensive care unit and started on 3% sodium chloride infusion. Brain magnetic resonance imaging (MRI) with contrast demonstrated heterogeneous, predominantly cystic expansion of the pituitary gland and distal infundibulum measuring 1.1 × 1.8 × 1.5 cm, with a nodular T2-hypointense and T1-hyperintense focus along the inferior pituitary margin (Fig. 1A–1B). Findings were most consistent with a Rathke cleft or pars intermedia cyst, with craniopharyngioma or hemorrhagic macroadenoma considered less likely. Neurosurgery recommended no surgical intervention, while nephrology advised continued hypertonic saline and initiation of tolvaptan for presumed SIADH.

Despite infusion of a 1600 mL of 3% saline over 36 hours, plasma sodium continued to decline, reaching a nadir of 114 mEq/L. The refractory hyponatremia and persistent symptoms prompted reconsideration of the diagnosis. Endocrinology raised pituitary apoplexy as a diagnostic consideration—based on the worsening headache and sellar pathology—and recommended empiric hormone replacement with IV hydrocortisone (100 mg every 6 hours) and levothyroxine (100 mcg daily) while awaiting pituitary and adrenal testing results.

## Outcome and follow-up

**Figure 2:**
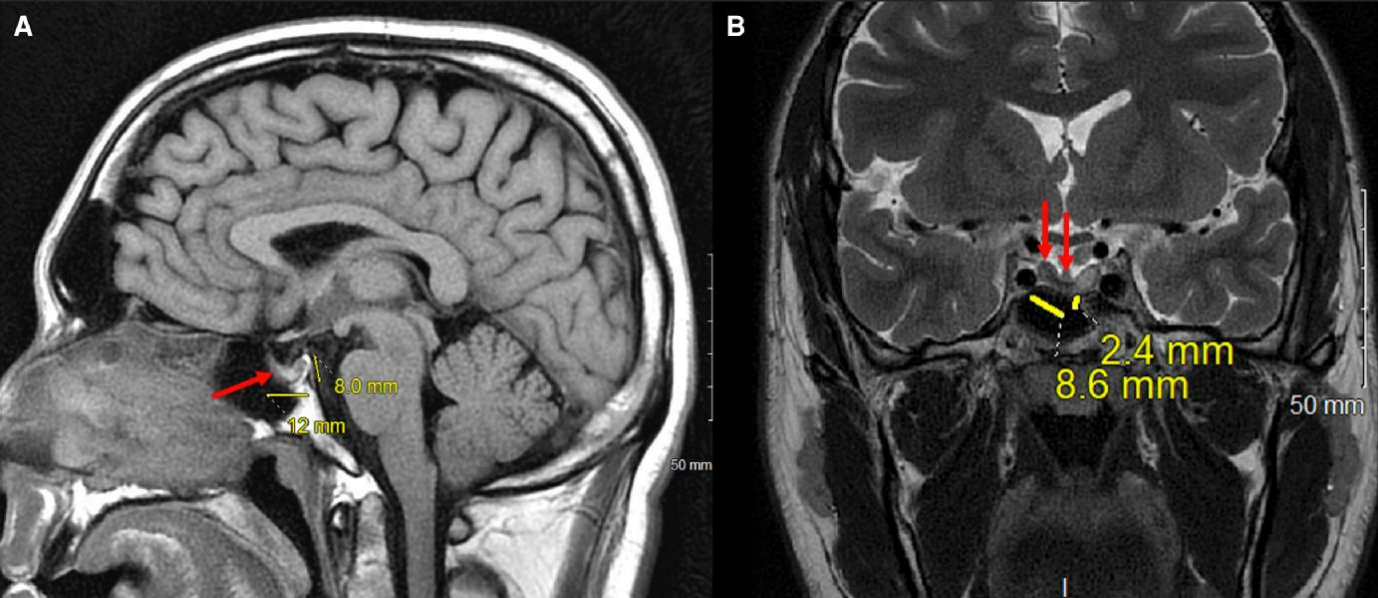
Subsequent magnetic resonance imaging of the brain with and without IV contrast. (A) Sagittal T1 view. (B) Coronal T2 view. The cyst (arrows in Fig. 2A) has decreased in size with mild TI hyperintensity focus. Hemorrhagic or proteinaceous debris is more clearly defined (identified by the arrows in Fig. 2B), and this can be a result of autoinfarction or involution of an adenoma consistent with response to treatment.

Following initiation of hormone replacement, his symptoms rapidly improved, and plasma sodium increased to 126 mEq/L (126 mmol/L) by discharge, with complete normalization on outpatient testing 1 week later (Table 1). He was discharged on hospital day 6 with a 3-day hydrocortisone taper, followed by hydrocortisone 15 mg each morning and 10 mg each afternoon and levothyroxine 125 mcg daily. At follow-up, his headaches had resolved. Repeat pituitary magnetic resonance imaging with and without contrast demonstrated interval reduction of the cystic size to 9 × 2 mm (Fig. 2B), but there were also cystic features of hemorrhagic and proteinaceous debris and T1 hyperintensity. These findings were characteristic of a hemorrhagic pituitary adenoma with cystic features that had significant expansion initially but then involuted from treatment. After 6 months of steroid and thyroxin replacement, recovery of his pituitary axes was observed, except for persistent GH deficiency (Table 2).

**Table 1:** Sodium and urine electrolyte trend during patient's hospitalization

	Hospital days					Outpatient
	Day 1 (admitted in the early evening)	Day 2	Day 3 (hypertonic saline started)	Day 4 (therapy started)	Day 6	1-week follow-up
Sodium						
136-145 mEq/L	123 mEq/L	122 mEq/L	120 mEq/L	118 at 0016	126 mEq/L at 1440	145 mEq/L
(136-145 mmol/L)				114 at 1619		
	(123 mmol/L)	(122 mmol/L)	(120 mmol/L)	123 at 2358	(126 mmol/L)	(145 mmol/L)
				(all values in mEq/L or mmol/L)		
Serum osmolarity						
270-295 mos/kg	260 mos/kg	-	-	-	-	-
270-295 mmol/kg	(260 mmol/kg)					
Urine osmolarity						
280-1200 mos/kg	575 mos/kg	-	-	962 mos/kg	-	-
280-1200 mmol/kg	(575 mmol/kg)			(962 mmol/kg)		
Uric acid						
3.5-7.2 mg/dL	-	5.3 mg/dL	-	3.5 mg/dL	-	-
μmol/L		(0.31 μmol/L)		(0.21 μmol/L)		
Urine sodium	-		-		-	-
No reference range		55 meq/L		143 meq/L		
Urine potassium	-		-		-	-
No reference range		29.3 meq/L		137.2 meq/L		
Urine chloride	-		-		-	-
No reference range		63.0 meq/L		213.0 meq/L		

On day 3, the patient was started on 3% normal saline for worsening symptoms and hyponatremia. His condition continued to worsen into day 4. It significantly improved in the evening of day 4 when the patient was started on hydrocortisone 100 mL IV every 6 hours and levothyroxine 100 mcg IV. His urine osmolarity also improved, demonstrating the importance of cortisol deficiency severely limiting free water excretion. Values in parentheses are in International System of Units.

**Table 2:** Pituitary and adrenal laboratory evaluation from hospitalization to outpatient monitoring

	Hospital days				Outpatient follow-up visits						
	Day 2	Day 3	Day 4 (start of therapy)	Day 6	1-week	1 month	3 month	4 month	6 month (end of therapy)	7 month	8 month
TSH											
0.35-4.94 μIU/mL	0.2 μIU/mL	-	0.078 μIU/mL	0.84 μIU/mL	0.47 μIU/mL	0.29 μIU/mL	0.69 μIU/mL	0.38 μIU/mL	0.54 μIU/mL	2.52 μIU/mL	1.93 μIU/mL
(0.35-4.94 mIU/L)	(0.2 mIU/L)		(0.078 mIU/L)	(0.84 mIU/L	(0.47 mIU/L)	(0.29 mIU/L)	(0.69 mIU/L)	(0.38 mIU/L)	(0.54 mIU/L)	(2.52 mIU/L)	(1.93 mIU/L)
Free T4											
0.70-1.48 ng/dL	0.82 ng/dL	-	1.50 ng/dL	0.84 ng/dL	1.36 ng/dL	1.23 ng/dL	1.15 ng/dL	1.34 ng/dL	1.18 ng/dL	0.86 ng/dL	1.3 ng/dL
(9.01-19.05 pmol/L)	(10.56 pmol/L)		(8.75 pmol/L)	(10.81 pmol/L)	(17.51 pmol/L)	(15.83 pmol/L)	(14.80 pmol/L)	(17.25 pmol/L)	(15.19 pmol/L)	(11.07 pmol/L)	(16.73 pmol/L)
T3											
0.58-1.59 ng/mL	-	-	0.52 ng/mL	0.64 ng/mL	-	-	0.89 ng/mL	0.85 ng/mL	-	-	-
(0.89 -2.44 nmol/L)			(0.80 nmol/L)	(0.98 nmol/L)			(1.36 nmol/L)	(1.31 nmol/L)			
Am cortisol*^a^*											
4.0-22.0 μg/dL	-	2.70 μg/dL at 1741	4.20 μg/dL at 0407	-	-	-	15.7 μg/dL at 0820	20.0 μg/dL at 0841	18.1 μg/dL at 0839	19.2 μg/dL at 0950	20.2 μg/dL at 0937
(110.3-606.9 nmol/L		(74.5 nmol/L)	(115.9 nmol/L)				(433.1 nmol/L)	(551.7 nmol/L)	(499.3 nmol/L)	(529.7 nmol/L)	(557.2 nmol/L)
ACTH											
6.0-50.0 pg/mL	-	-	-	<5 pg/mL	-	-	26 pg/mL	23 pg/mL	19 pg/mL	21 mg/mL	23 mg/mL
(1.3-11.0 pmol/L)				(<1.1 pmol/L)			(5.7 pmol/L)	(5.1 pmol/L)	(4.6 pmol/L	(4.6 pmol/L)	(5.1 pmol/L)
Testosterone											
250-1100 ng/dL	-	22 ng/dL	-	-	309 ng/dL	350 ng/dL	-	-	-	-	-
(8.67-34.7 nmol/L)		(0.76 nmol/L)			(10.7 nmol/L)	(12.1 nmol/L)					
IGF-1											
83-456 ng/mL	-	321 ng/mL	-	-	272 ng/mL	-	-	-	-	-	—
(10.9-59.6 nmol/L)		(42.0 nmol/L))			(37.9 nmol/L)						
GH											
< or = 7.1 ng/mL	-	0.4 ng/mL	-	-	0.7 ng/mL	0.1 ng/mL	-	-	-	-	-
(< or = 7.1 μg/L)		(0.4 μg/L)			(0.7 μg/L)	(0.1 μg/L)					
Prolactin											
2.1-17.7 ng/mL	8.0 ng/mL	6.6 ng/mL	-	-	9.1 ng/mL	-	-	-	-	-	-
(2.1-17.7 μg/L)	(8.0 μg/L)	(6.6 μg/L)			(9.1 μg/L)						
FSH											
1.6-8.0 mIU/mL	-	1.50 mIU/mL	-	-	5.60 mIU/mL	5.2 mIU/mL	-	-	-	-	-
(1.6-8.0 IU/L)		(1.50 IU/L)			(5.60 IU/L)	(5.2 IU/L)					
LH											
1.5-9.3 mIU/mL	-	0.62 mIU/mL	-	-	4.4 mIU/mL	3.7 mIU/mL	-	-	-	-	-
(1.5-9.3 IU/L)		(0.62 IU/L)			(4.4 IU/L)	(3.7 IU/L)					

Patient was started on hydrocortisone 100 mL IV every 6 hours and levothyroxine 100 mcg IV daily on day 4 of hospitalization. He was discharged with an oral taper of the hydrocortisone stress dose followed by hydrocortisone 15 mg in the morning and 10 mg in the afternoon and levothyroxine 125 mcg oral every morning. Patient received a 6-month total duration of treatment. Values in parentheses are in International System of Units.

^*a*^To be drawn between 7 and 9 Am.

## Discussion

This case describes pituitary apoplexy presenting with severe, refractory euvolemic hypotonic hyponatremia initially attributed to SIADH. Despite conventional SIADH therapy, plasma sodium continued to decline, while rapid improvement followed glucocorticoid replacement. This highlights the diagnostic challenge of distinguishing pituitary apoplexy from more common causes of euvolemic hyponatremia and the importance of recognizing secondary adrenal insufficiency, especially in progressive or treatment-resistant cases.

At presentation, the patient had hypotonic hyponatremia without clinical or laboratory evidence of hypovolemia or hypervolemia, narrowing the differential to euvolemic causes. Urine osmolality was higher than expected for the degree of plasma hypotonicity, arguing against conditions with intact water excretion (eg, primary polydipsia or low solute intake) and with no medication exposure to suggest a drug-related cause. Hypotonic hyponatremia with inappropriately concentrated urine and apparent euvolemia therefore supported a preliminary diagnosis of SIADH [1, 4]. Although central hypothyroidism was present and considered a potential contributor, it was unlikely to account for the severity or progression of hyponatremia. However, the diagnosis was questioned when plasma sodium continued to decline despite the administration of 3% saline—which has a sodium concentration nearly twice that of maximally concentrated urine—along with worsening symptoms and increasing urine output, suggesting a process more complex than straightforward SIADH.

In this context, the presence of a sellar lesion in the setting of treatment-refractory hyponatremia suggested pituitary-related disruption of water homeostasis. Pituitary and hypothalamic pathology can produce an SIADH-like biochemical pattern, though the underlying physiology is different [5-7]. Several mechanisms can contribute. Central hypothyroidism can impair free-water excretion through reduced cardiac output and glomerular filtration; however, this effect is usually mild, develops gradually, and is unlikely to explain severe or treatment-resistant hyponatremia [8]. Attention therefore shifts to antidiuretic hormone (ADH) regulation. Because ADH is synthesized in hypothalamic nuclei and released via the posterior pituitary, pathology involving the pituitary or infundibulum can disrupt hypothalamic–pituitary signaling and result in inappropriate ADH release not suppressed by hypotonicity [9]. In addition, acute stressors such as pain, nausea, and central nervous system injury can trigger nonosmotic ADH release [9]. Finally, acute secondary adrenal insufficiency represents a particularly important mechanism by which pituitary pathology can masquerade as SIADH, as cortisol deficiency removes tonic inhibition of ADH secretion, leading to sustained ADH activity, impaired free-water excretion, and euvolemic hyponatremia [5, 7, 9, 10]. Together, these processes explain an SIADH-like profile that does not respond to conventional therapy.

Against this clinical and physiologic backdrop, pituitary apoplexy provided a unifying explanation for the patient's presentation and course. Acute worsening headache in the setting of a sellar lesion, followed by rapidly progressive and treatment-refractory hyponatremia, is consistent with sudden disruption of hypothalamic–pituitary function [2, 3, 5]. This disruption can lead to central dysregulation of ADH release and precipitate secondary adrenal insufficiency, thereby exacerbating water retention and reducing the effectiveness of SIADH-directed therapies. Rapid improvement in plasma sodium and symptoms following glucocorticoid replacement further supports pituitary apoplexy as the principal driver of hyponatremia rather than a primary disorder of ADH secretion [5].

The effects of pituitary apoplexy on sodium–water homeostasis arise from multiple converging mechanisms acting simultaneously. Acute hemorrhage or infarction within the pituitary can impair hypothalamic–pituitary signaling along the pituitary stalk, leading to dysregulated ADH release [2, 3]. Because ADH is synthesized in hypothalamic nuclei, stalk compression or ischemia may impair inhibitory control and result in persistent, centrally mediated ADH activity. In parallel, pituitary apoplexy frequently precipitates secondary adrenal insufficiency due to loss of corticotropin secretion. Cortisol normally tonically inhibits ADH release and supports renal free-water excretion effects on glomerular filtration and distal tubular water handling; cortisol deficiency therefore results in unopposed ADH activity and impaired free-water excretion [5, 9, 10]. Nonosmotic ADH stimulation from headache, nausea, and intracranial stress during the apoplectic event may further augment water retention. Together, these mechanisms account for the progressive, treatment-refractory hyponatremia observed, while the rapid response to glucocorticoid replacement implicates cortisol deficiency as the dominant reversible contributor.

Several features prompted consideration of salt wasting as a contributor to refractory hyponatremia, including discordance in urine electrolyte findings, a higher-than-expected urine output for SIADH, disproportionate increases in urine output following saline administration, and continued decline in plasma sodium despite hypertonic saline. While increased natriuresis and urine output would be expected in the setting of hypotonicity and repeated sodium loading, the magnitude of these responses appeared atypical. Unfortunately, the absence of timed urine volume and electrolyte collections precluded calculation of electrolyte-free water excretion and assessment for salt wasting. Moreover, despite the inherent imprecision of clinical volume assessment, there was no evidence of volume depletion or hemodynamic instability, without which salt wasting—renal or cerebral—cannot be diagnosed [11, 12]. Nonetheless, the clinical picture suggests that disordered water handling, rather than salt wasting, is the primary driver of hyponatremia.

Pituitary apoplexy is an uncommon, heterogeneous syndrome caused by hemorrhage or infarction of pituitary tissue, most often but not exclusively in the setting of pituitary adenomas [2, 3]. Although adenomas are a recognized risk factor, apoplexy occurs in only a subset of patients, with reported incidence ranging from approximately 1% to 26%. A slight male predominance has been described, and presentation most commonly occurs in the fourth decade of life or later, though cases outside these demographic patterns are well recognized. Additional precipitating factors such as trauma or intracranial infection have been reported, but many patients present without an identifiable trigger. Early manifestations are frequently nonspecific, and metabolic abnormalities may predominate over classic neuro-ophthalmologic findings, delaying recognition. As illustrated by this case, hyponatremia may be the dominant presenting feature and can closely resemble SIADH biochemically. However, elevated ADH levels in this setting reflect secondary hypothalamic–pituitary disruption—particularly cortisol deficiency—rather than primary SIADH, explaining why hyponatremia may persist or recur until cortisol deficiency is recognized and corrected [5, 6].

In summary, pituitary apoplexy is a rare but potentially life-threatening condition whose presentation may include prominent metabolic abnormalities alongside neurologic features. This case underscores the importance of considering pituitary pathology in patients with refractory hyponatremia, particularly by neurologic symptoms or evidence of hypothalamic–pituitary axis dysfunction. The hyponatremia is driven by secondary adrenal insufficiency resulting from pituitary injury, making timely recognition of pituitary apoplexy and its associated cortisol deficiency essential for effective treatment.

## Learning points

- Pituitary apoplexy may present with severe, refractory euvolemic hyponatremia even in the absence of classic neuro-ophthalmologic findings.
- Hyponatremia associated with pituitary apoplexy often reflects secondary adrenal insufficiency and central dysregulation of ADH rather than true SIADH.
- Early recognition of cortisol deficiency and prompt glucocorticoid replacement are essential to reversing hyponatremia and preventing further clinical deterioration.

## Contributors

All authors made individual contributions to this work. S.C. and S.Ta. were involved in the diagnosis and management of the patient. S.C. and T.B. contributed to data collection and preparation of the case report. S.Ta. and S.Th. contributed to the development of the discussion section. L.A.J. reviewed all available case information and played a central role in drafting and refining the final manuscript. All authors reviewed and approved the final version of the manuscript.

## Data Availability

Data sharing is not applicable to this article as no datasets were generated or analyzed during the current study.
